# Identifying key questions in the ecology and evolution of cancer

**DOI:** 10.1111/eva.13190

**Published:** 2021-02-08

**Authors:** Antoine M. Dujon, Athena Aktipis, Catherine Alix‐Panabières, Sarah R. Amend, Amy M. Boddy, Joel S. Brown, Jean‐Pascal Capp, James DeGregori, Paul Ewald, Robert Gatenby, Marco Gerlinger, Mathieu Giraudeau, Rodrigo K. Hamede, Elsa Hansen, Irina Kareva, Carlo C. Maley, Andriy Marusyk, Nicholas McGranahan, Michael J. Metzger, Aurora M. Nedelcu, Robert Noble, Leonard Nunney, Kenneth J. Pienta, Kornelia Polyak, Pascal Pujol, Andrew F. Read, Benjamin Roche, Susanne Sebens, Eric Solary, Kateřina Staňková, Holly Swain Ewald, Frédéric Thomas, Beata Ujvari

**Affiliations:** ^1^ School of Life and Environmental Sciences Centre for Integrative Ecology Deakin University Waurn Ponds Vic. Australia; ^2^ CREEC/CANECEV, MIVEGEC (CREES), University of Montpellier, CNRS, IRD Montpellier France; ^3^ Biodesign Institute Department of Psychology Arizona State University Tempe AZ USA; ^4^ Laboratory of Rare Human Circulating Cells (LCCRH) University Medical Center of Montpellier Montpellier France; ^5^ Brady Urological Institute The Johns Hopkins School of Medicine Baltimore MD USA; ^6^ Department of Anthropology University of California Santa Barbara Santa Barbara CA USA; ^7^ Department of Integrated Mathematics Moffitt Cancer Center Tampa FL USA; ^8^ Toulouse Biotechnology Institute INSA/University of Toulouse CNRS INRAE Toulouse France; ^9^ Department of Biochemistry and Molecular Genetics Integrated Department of Immunology Department of Paediatrics Department of Medicine (Section of Hematology) University of Colorado School of Medicine Aurora CO USA; ^10^ Department of Biology University of Louisville Louisville KY USA; ^11^ Department of Radiology H. Lee Moffitt Cancer Center & Research Institute Tampa FL USA; ^12^ Translational Oncogenomics Lab The Institute of Cancer Research London UK; ^13^ Littoral Environnement et Sociétés (LIENSs) UMR 7266 CNRS‐Université de La Rochelle La Rochelle France; ^14^ School of Natural Sciences University of Tasmania Hobart TAS Australia; ^15^ Center for Infectious Disease Dynamics, Biology Department Pennsylvania State University University Park PA USA; ^16^ Mathematical and Computational Sciences Center School of Human Evolution and Social Change Arizona State University Tempe AZ USA; ^17^ Arizona Cancer Evolution Center Biodesign Institute and School of Life Sciences Arizona State University Tempe AZ USA; ^18^ Department of Cancer Physiology H Lee Moffitt Cancer Centre and Research Institute Tampa FL USA; ^19^ Translational Cancer Therapeutics Laboratory The Francis Crick Institute London UK; ^20^ Cancer Research UK Lung Cancer Centre of Excellence University College London Cancer Institute London UK; ^21^ Pacific Northwest Research Institute Seattle WA USA; ^22^ Department of Biology University of New Brunswick Fredericton NB Canada; ^23^ Department of Biosystems Science and Engineering ETH Zurich Basel Switzerland; ^24^ Department of Evolutionary Biology and Environmental Studies University of Zurich Zurich Switzerland; ^25^ Department of Evolution, Ecology, and Organismal Biology University of California Riverside Riverside CA USA; ^26^ Department of Medical Oncology Dana‐Farber Cancer Institute Boston MA USA; ^27^ Department of Medicine Harvard Medical School Boston MA USA; ^28^ Centre Hospitalier Universitaire Arnaud de Villeneuve Montpellier France; ^29^ Center for Infectious Disease Dynamics Huck Institutes of the Life Sciences Departments of Biology and Entomology Pennsylvania State University University Park PA USA; ^30^ Unité Mixte Internationale de Modélisation Mathématique et Informatique des Systèmes Complexes UMI IRD/Sorbonne Université UMMISCO Bondy France; ^31^ Institute for Experimental Cancer Research Kiel University and University Hospital Schleswig‐Holstein Kiel Germany; ^32^ INSERM U1287 Gustave Roussy Villejuif France; ^33^ Faculté de Médecine Université Paris‐Saclay Le Kremlin‐Bicêtre France; ^34^ Department of Data Science and Knowledge Engineering Maastricht University Maastricht The Netherlands; ^35^ Delft Institute of Applied Mathematics Delft University of Technology Delft The Netherlands

**Keywords:** cancer therapy, contemporary evolution, evolutionary medicine, genetics, neoplasm, species interactions

## Abstract

The application of evolutionary and ecological principles to cancer prevention and treatment, as well as recognizing cancer as a selection force in nature, has gained impetus over the last 50 years. Following the initial theoretical approaches that combined knowledge from interdisciplinary fields, it became clear that using the eco‐evolutionary framework is of key importance to understand cancer. We are now at a pivotal point where accumulating evidence starts to steer the future directions of the discipline and allows us to underpin the key challenges that remain to be addressed. Here, we aim to assess current advancements in the field and to suggest future directions for research. First, we summarize cancer research areas that, so far, have assimilated ecological and evolutionary principles into their approaches and illustrate their key importance. Then, we assembled 33 experts and identified 84 key questions, organized around nine major themes, to pave the foundations for research to come. We highlight the urgent need for broadening the portfolio of research directions to stimulate novel approaches at the interface of oncology and ecological and evolutionary sciences. We conclude that progressive and efficient cross‐disciplinary collaborations that draw on the expertise of the fields of ecology, evolution and cancer are essential in order to efficiently address current and future questions about cancer.

## 1 INTRODUCTION

The application of evolutionary and ecological principles to preventing and treating cancer (Gatenby & Brown, 2018), as well as to understanding the impact of cancer on organismal health, fitness, species stability and ecosystem functioning (Thomas et al., 2017), has been gaining increasing attention and recognition among both oncologists and biologists since the seminal work of Cairns (1975), Nordling (1953) and Nowell (1976), more than 45 years ago. Most scientists today agree that this evolutionary view has deeply transformed the way we understand the biology of cancer—explaining its origin and the recrudescence of cancer cells as well as elucidating reasons for therapy failures. Following the theoretical development of a new interdisciplinary field that combines expertise from mathematicians, data scientists and biostatisticians, geneticists, evolutionary biologists, ecologists, physicists and oncologists, we are now at a pivotal point where empirical data and evidence are accumulating and guiding future directions of the discipline (Ujvari et al., 2017). We believe that the time has arrived to take stock of current advancements and to inform the course of future research. Cancer is a disease that impacts every country worldwide (18.1 million new cases and 9.6 million death in 2018; Bray et al., 2018), and these oncogenic processes are an inevitable phenomenon of metazoan life. Identifying the key questions in the ecology and evolution of cancer will provide a cornerstone in cancer and evolutionary research for the coming years. This will provide the basis for the development of efficient strategies to either prevent cancer evolution or improve treatment of even advanced cancers.

A recently published viewpoint article presents a valuable roadmap for the next decade in cancer research (Bernards et al., 2020). However, this roadmap, based on the opinion of 10 researchers, does not mention how the ecological and evolutionary theory, principles and approaches have already provided major and novel insights into our understanding of several cancer‐related topics, nor provides future avenues of research studying cancer with an evolutionary biology approach. Below, we first summarize the main cancer‐related areas that benefited from applying ecological and evolutionary thinking. Then, we identified and highlighted key questions, and organized around nine major themes based on the systematic classification and ranking of the feedback obtained from the 33 scientists that contributed to this study (see section 2).

### 1.1 Cancer as a complex eco‐evolutionary process

Neoplasia has been detected in most multicellular groups, suggesting that its evolutionary roots can be traced back to the evolution of multicellularity (Ackermann, 2015). In fact, cancer is often seen as a by‐product of multicellularity, specifically a breakdown of the mechanisms that evolved to ensure the functionality of the newly emerged multicellular individual by promoting cooperation among constituent cells (Aktipis et al., 2015). In this framework, cancer cells are selfish/cheater cells whose success is dependent on the failure of the multicellular organisms to suppress, detect and police them (Aktipis et al., 2015; Aktipis, 2020). Differences in the propensity to develop cancer among species can thus be understood not only as the result of differences in mutation hazard (intrinsic or extrinsic) but also as the result of differences in the ability to prevent and deal with such selfish mutants (i.e. differences in tumour suppression mechanisms). Following the acknowledgement that oncogenic processes are inevitable phenomena in all metazoans since the dawn of multicellularity, the field of comparative oncology—the study of oncology in non‐human organisms—has brought relevant insights into how biological, genetic and ecological factors drive individual and species variations in cancer diversity, incidence, therapy resistance and lethality. As such, it opens the opportunity to develop a universal theory of cancer biology that promises to revolutionize conventional preclinical models and cancer treatment strategies (Albuquerque et al., 2018; Somarelli et al., 2020).

### 1.2 Understanding cancer's evolutionary history

Each of the nearly 10 million people dying from cancer every year developed that lethal cancer de novo (Bray et al., 2018; Pienta et al., 2020; Siegel et al., 2018). Over the last 50 years, a series of mutually non‐exclusive but concurrent theories have been put forward to explain the initiation and progression of cancer. (a) The classic model of stepwise carcinogenesis, first proposed by Nordling (Nordling, 1953) in 1953 and then by Nowell (1976) in 1976, posits that a transformed cell gains unlimited proliferative capacity and uncontrolled cell growth via subsequent accumulation of random mutations. Once a heterogeneous cell subpopulation is initiated within the tissue environment, natural selection favours cancer cells harbouring mutations that confer higher fitness, making these cell clones the most prominent in the population. The recurring cycles of clonal sweeps lead to cancer growth, progression and dispersal (i.e. metastasis). (b) Similar to the classic model, the hierarchical model (Costa et al., 2006; Wicha et al., 2006) also traces tumour origins to single mutated cells with unlimited proliferative potential, but assumes that the development of the tumour results from the clonal evolution of cells with stem cell properties (Lapidot et al., 1994; Sell, 1993; Tan et al., 2006; Visvader & Lindeman, 2008). Independent of the type of cancer progenitor cells (i.e. somatic cells or cancer stem cells), both theories portray cancer progression as the accumulation of genetic modifications (mutations and epigenetic alterations) and expansion of clones with higher fitness.

While the early models proposed gradual accumulation of genomic alterations to acquire the selective advantages by the malignant cells (Fearon & Volgelstein, 1990), later, karyotype‐based studies suggested a stochastic cancer evolution model, with cancer cell populations alternating between punctuated (rapid, stochastic karyotype changes) and sequential phases (subsequent clonal expansion of cancer cells) (Yates & Campbell, 2012).

The study of metastasis has also benefited from eco‐evolutionary thinking. The movement of malignant cells from the primary tumour to a secondary site in the host's body is likely in response to the selective pressure within the tumour microenvironment, including resource scarcity, increased risk of death and overcrowding (Aktipis et al., 2012; Chen et al., 2011). While being highly risky, this migration significantly increases the fitness of malignant cells, which allows scientists to draw parallels with the way animals migrate and disperse to increase their fitness (Aktipis et al., 2012; Chen et al., 2011; Tissot et al., 2019).

In 1889, Steven Paget proposed the ‘Seed and Soil’ hypothesis, introducing the concept that a receptive microenvironment was required for malignant cells to engraft distant tissues and form metastases (Paget, 1889). It is now well established that the processes of clonal cell expansion, diversification and selection that characterizes malignant tumour evolution occur within the tissue ecosystem and microenvironment, and include the selective pressure generated by the treatment which contributes for the selection of resistant variants (Chen & Pienta, 2011). Cancer cells themselves alter their microenvironment to their own benefit, by promoting angiogenesis, changing the functions of stromal cells, inducing neural damage, neutralizing immune cells and promoting an immunosuppressive environment (Costa et al., 2018).

### 1.3 Applying eco‐evolutionary principles to manage and treat cancer

Theodosius Dobzhansky famously stated that ‘nothing in biology makes sense except in the light of evolution’ (Dobzhansky, 1973). The emergence of cancerous cells can be seen as a speciation event, in which a new parasitic species emerges, initiates a clade and consumes resources from the host, impairing the host's health and decreasing its fitness (Capp & Thomas, 2020; Duesberg et al., 2011). When a cancer emerges, its progression is then governed through Darwinian selection (referred as somatic evolution) that is separate from the host (the unit of natural selection). In addition, a tumour can be considered as being a whole ecosystem in which cells adapt and evolve to exploit the resources of the environment or to develop resistance to drug treatment (Aktipis & Nesse, 2013; Gatenby & Brown, 2018; Nowell, 1976). It is therefore of key importance to understand the coevolutionary dynamics of cancer functioning to design efficient treatments and improve the outcome of patients. Such understanding may be enhanced by extending concepts (e.g. commensalism, parasitism, predation, ecological niche, selective pressure) or tools such as population dynamics or game theory models widely used in ecology (Archetti & Pienta, 2019; Dhawan et al., 2016; Maley et al., 2017) to treatment approaches and strategies. Adaptive therapy, in which cancer is treated by alternating different drugs to avoid selection for resistant cancer cells, is a primary example of the successful application of the theory of evolution in cancer treatment (Gatenby et al., 2009).

### 1.4 A general view on the interaction between species evolution and cancer incidence

Oncogenic processes, and the resulting selection of costly host defences yielding to trade‐offs, have been a major force shaping ecological and evolutionary processes in the animal kingdom (Aktipis & Nesse, 2013; Thomas et al., 2017). This especially applies to transmissible cancers, which can threaten the survival of species and raise the question of the extent to which they can be considered to be a separate species from their hosts (Russell et al., 2018). Just as with other animals, the evolution of humans was likely shaped by cancer (Boutry et al., 2020; Kang & Michalak, 2014; Thomas, Giraudeau, Renaud, et al., 2019). The environments in which humans now live and the associated lifestyle changes have undergone dramatic alterations since prehistoric times (Greaves & Aktipis, 2016). Humans have been living to older ages (Gurven & Kaplan, 2007), and incidences of cancer have therefore also increased (Nesse, 2005). Since most cases of cancers exert their negative effect on survival in the post‐reproductive stage, the effect of fitness is often minimal so that natural selection will be rather ineffective at decreasing cancer's negative impact. Combining the knowledge of comparative oncology with the evolutionary ecology principles of multicellular organisms and ecosystem functioning will have implications for not only cancer treatment but also conservation biology given our changing, often increasingly polluted, world (Dujon et al., 2020; Hamede et al., 2020). Thus, the interdisciplinary field of research, *Ecology, Evolution and Cancer*, is not only transforming our understanding of cancer and the strategies to prevent and to cure it, but it also sheds light on the major influence of oncogenic processes on the interactions between biotic and abiotic components of ecosystems.

## 2 METHODS

**FIGURE 1:**
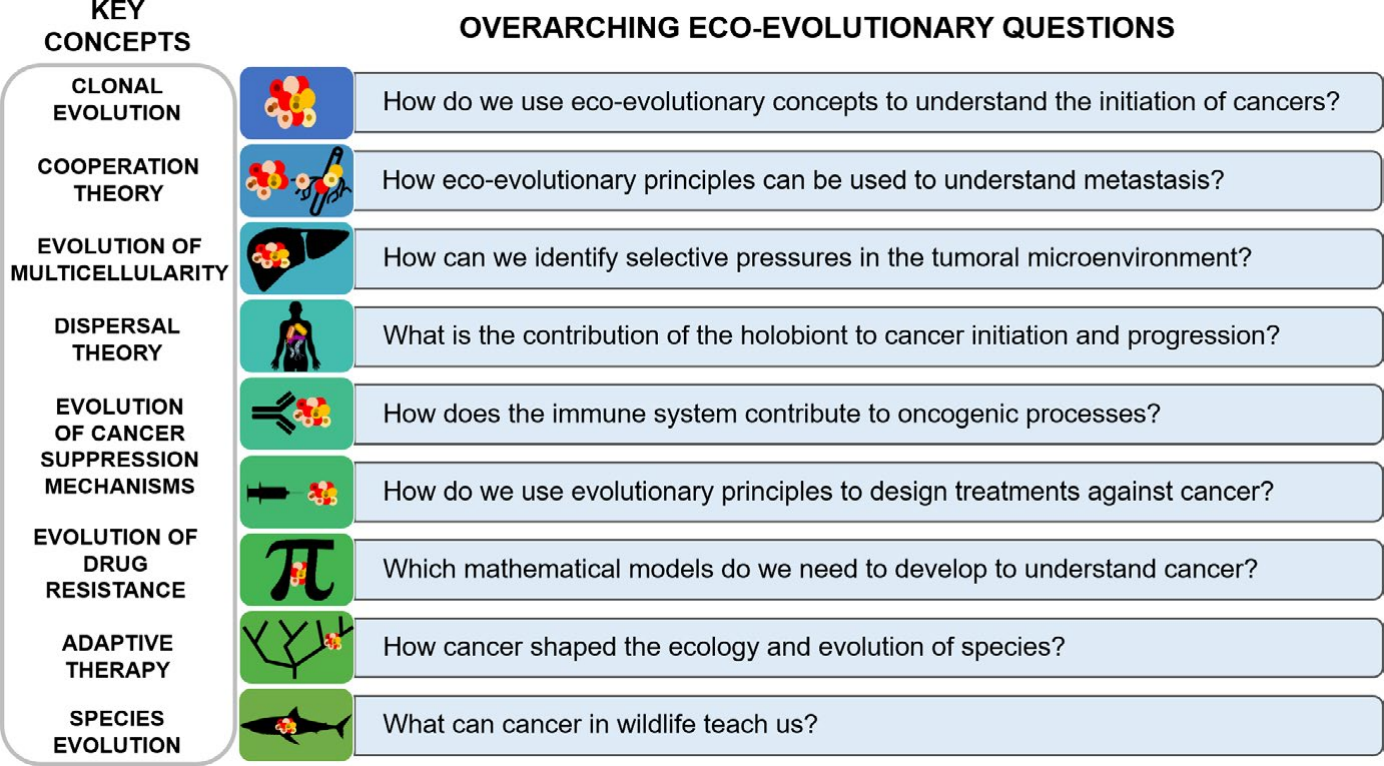
The nine overarching eco‐evolutionary questions based on key ecological and evolutionary concepts to answer in the years to come to obtain new insights on cancer

We adapted the protocol previously used to identify 100 fundamental questions in ecology (Sutherland et al., 2013). We initially identified and selected established leading experts in the field based on their publication records and the extent of their work in the study of ecology and evolution of cancer, and contacted them by email. In addition, the participants were allowed to suggest additional experts who were also selected based on the same criteria. Each participant was invited to provide a list of the five most fundamental questions of the respective discipline, ranked by decreasing importance. Each participant also provided a short highlight of up to 150 words, and relevant references, detailing why each question is of key importance for the field. All questions from the participants were then compiled by three independent researchers, and similar responses were processed into a single question. Then, the most common questions from this list were identified and reduced to a total of 84 which were grouped into nine major themes (Figure 1). The nine major themes were first defined by the three researchers that compiled the responses to the questions and are presented in order of increasing spatial scales, ranging from the size of a cell up to whole ecosystems. The nine themes were then sent to all authors to be validated. In addition, a small literature review was also created for each of the nine themes based on the expert summaries provided by the respondents. The full list of questions is provided as Appendix S1.

## 3 RESULTS

### 3.1 Major theme 1: Cancer initiation and progression

The recent comprehensive genomic characterization of tumours by the Pan‐Cancer Analysis of Whole Genomes (PCAWG) Consortium, a great example of efficient scientific collaboration, gave insights into some of the underlying mechanisms driving cancer initiation and progression (Campbell et al., 2020; Cieslik & Chinnaiyan, 2020), and potentially provides support for the ‘punctuated and stepwise evolution’ theory. Briefly, PCAWG demonstrated chromothripsis (when clustered structural variants arise in a single catastrophic event) to frequently be an early event in tumour evolution (see Campbell et al., 2020 for proportion details across a range of cancer type), and highlighted the importance of driver mutations, in both coding and non‐coding regions (Campbell et al., 2020; Li et al., 2017; Rheinbay et al., 2020). By applying molecular clocks to classify clonal and subclonal mutations, Gerstung et al. (2020) found that highly recurrent driver mutations and copy‐number gains in particular tumour types tend to occur the earliest in a given cancer type (Li et al., 2017), and they also tend to precede diagnosis by many years, if not decades. PCAWG also identified characteristic genomic aberrations, called signatures, arising from defective DNA‐repair mechanisms or exposure to environmental mutagens (Alexandrov et al., 2020; Gerstung et al., 2020).

These recent large‐scale genome and transcriptome studies of tumour genomes clearly show that cancer initiation is not a step‐by‐step process simply driven by the sequential accumulation of mutations but rather a dynamic evolutionary process that depends on microenvironmental and complex genomic and epigenetic landscapes. As proposed by Gatenby et al. (2020), cells in multicellular organisms are involved in the cooperative functioning of the organism and the host is the unit of natural selection. Once mutations accumulate, cancer cells are able to abrogate control by local tissue constraints and become free from host constraints, and their newly acquired individual fitness is determined by the Darwinian interactions of their phenotype with critical properties of their local environment. Mutations previously accumulated over the lifetime of the host serve as their genetic heritage in their malignant trajectory and allow for somatic selection to favour the most adapted cell lines (e.g. to their local microenvironment).

Dynamic epistatic and pleiotropic processes further increase the enormous variation in cancer risk per (stem) cell division, both between tissues and between species (Caulin & Maley, 2011; Noble et al., 2015). Ultimately, cancer cells form tumours comprising different specialized cell populations (Aktipis et al., 2015); however, many cancer cells lose their original phenotype and acquire new functions. Apart from the diversity of tumour cell phenotypes (functions), tumours contain a plethora of non‐neoplastic cells which also contribute to division of labour (Barcellos‐Hoff et al., 2013). Overall, the fitness of cancer cell lineages in a tumour is seen as a collective rather than an individual achievement with an ability to evolve resistance to a number of known therapies (Capp, 2019; Lichtenstein, 2019).

As cancer cells are fast proliferating cheater cells that take advantage of the benefits of multicellular tissue (e.g. blood flow) without performing their original differentiated function for the host, cancer suppression systems (including the immune system) are sometimes been seen as cheater detection systems. Those systems prevent the emergence or limit the proliferation of cheater cells, for example by causing apoptotic response to DNA damage or inappropriate proliferation, the sequestration of rare stem cells or immune surveillance (Aktipis et al., 2015). By applying cooperation (game) theory to cancer biology (Archetti & Pienta, 2019), we can gain more leverage on questions about how cancer suppression systems work to prevent cancer initiation and progression (Aktipis et al., 2015) and how we can better support our innate cellular cheater detection systems to prevent cancer in the first place (Aktipis, 2020). Tumours are often detected years/decades after their initiation (see above), and overcoming this challenge will require improved understanding of conditions that are favourable to cancer initiation at the nascent stages of cheater‐cell emergence. Addressing the questions raised in this publication could strongly help to prevent cancer development. For example, the obtained insight could be used to develop treatments to reduce cancer initiation for people exposed to risk factors they cannot avoid.

#### 3.1.1 Questions

1. What is the cell of origin in cancers?
2. Can normal somatic cells evolve into cancer? Do somatic cells accumulate genetic mutations that would facilitate their adaptation to their local environment without crossing the threshold of becoming malignant?
3. How can mutant clones expand in normal tissues?
4. How can early cancer‐driving mutations be not eliminated from the host's genome when tumours start to evolve?
5. Which genetic aberrations acquired in subclones during cancer progression confer a fitness advantage?
6. How does division of labour drive tumour evolution?
7. How do epistatic interactions shape cancer development?
8. Is the inclusion of an ecological perspective required to understand initiation and progression of cancers?
9. Why do some organs develop on average more cancers than others?
10. Which mechanisms explain variation in cancer risk, relative to lifetime number of stem cell divisions?
11. Do particular life periods exist in which humans and animals are especially vulnerable to cancer initiation and why?
12. What is the core eco‐evolutionary programme that manifests independently in cancer patients leading to death?
13. Can we perform an extensive evaluation of intrinsic factors involved in clonal cancer evolution? Can we gain a better understanding of the clonal evolution of malignant cells by an applying systems biology approach to understand the interactions between intrinsic factors?
14. How does host phenotypic plasticity (e.g. life‐history trait adjustments, compensatory responses) in response to oncogenic processes affect the fitness and evolution of cancer cells?
15. How our understanding of oncogenesis and cancer progression can be improved by applying evolutionary and developmental biology paradigms to cancer evolution?
16. What is the role of inter‐ and intra‐clonal competition or cooperation in cancer initiation?
17. How can we integrate different sources of phenotypic variability towards inclusive inheritance in initiation of cancer and somatic evolution?
18. Although specific cellular traits/markers distinguish benign from malignant tumours, what evolutionary processes determine the specific trajectories resulting in the two type of tumours?

### 3.2 Major theme 2: Cancer metastasis

Metastasis is the process involving the detachment of malignant cells from the primary tumour site, their dispersal within the body and their colonization of secondary sites. Cancer metastasis accounts for the overwhelming majority of cancer‐related deaths (>90%), and if cancer cells did not metastasize, the majority of all cancers could be cured by primary therapy (the first treatment given for a disease, e.g. surgery) (Chaffer & Weinberg, 2011). Despite arising independently in each patient, cancer progression often follows a similar eco‐evolutionary path, eventually manifesting as incurable lethal disease. Such convergent evolution across hundreds of thousands of metastatic cancer patients each year necessitates an eco‐evolutionary explanation beyond the typically cited acquisition of stochastic mutations giving rise to tumour cell heterogeneity. The movement of malignant cells from the primary tumour is considered to be a stochastic event (Chaffer & Weinberg, 2011; De Groot et al., 2017; Fidler, 2003; Pienta et al., 2013), with shedding of billions of cancer cells required to successfully establish new tumour(s) at a distant tumour site, as the majority of circulating cancer cells perishes, and only a very small minority of metastatic cells ultimately form a clinically apparent tumour (De Groot et al., 2017; Tissot et al., 2019). Using the theoretical and experimental insights obtained by ecologists and evolutionary biologists who have been working on dispersal and migration in plants and wildlife species provides the great opportunity to improve our understanding of the complex metastatic process (e.g. by drawing a parallel with species relying on a stochastic environment to disperse eggs, larvae and propagules) (Tissot et al., 2019). Deciphering the evolutionary principles driving early dissemination of oncogenic cells might help to develop strategies to prevent colonization of secondary sites, thereby minimizing metastases.

#### 3.2.1 Questions

19. Why do malignant cells metastasize since only a small fraction of cancer cells survives the metastatic cascade, and can we apply eco‐evolutionary principles to identify the conditions and factors that inaugurate the transitioning of a somatic cell to a cancer cell with affinity for dispersal?
20. What are the ecological characteristics of the tumour that induce dispersal from the primary tumour?
21. What is the role of the high tumour genetic heterogeneity during metastases formation?
22. What is a useful model to analyse the eco‐evolutionary dynamics of early metastatic development?
23. How can we use ecological principles such as those in biological control to suppress the initiation of the metastatic process?

### 3.3 Major theme 3: Tumour and microenvironment

The microenvironment of cancer cells is their ecology, and so concepts and tools from ecology are likely to be useful for the study of the tumour microenvironment. Coupling appropriate evolutionary game and ecological dynamics could potentially identify the absolute minimum resources cancer cells (and their population) need to survive, thus offering a novel avenue for therapies. Cancer cells alter their microenvironment, and recent data have substantiated the view that changes/evolution of the microenvironment is mechanistically linked to drug resistance (Hirata et al., 2015; Woolston et al., 2019). Furthermore, inflammation associated with changes during ageing promotes selection for cells with adaptive oncogenic phenotypes (Barcellos‐Hoff et al., 2013; Henry et al., 2015; Laconi et al., 2020). In addition, a number of experiments have highlighted the ability of a healthy microenvironment to repress oncogenic transformation through various mechanisms such as immune surveillance and maintenance of tissue structure via the extracellular matrix and healthy tissue (stroma) surrounding the tumour (Strobl et al., 2020).

However, no consensus has so far been drawn on how to define and spatially delineate the tumour environment that extends from the tumour to the whole organism, including associated microorganisms and toxic exposures (Laplane et al., 2018). Another conundrum that also remains is whether alterations of the microenvironment observed in cancer are secondary to cancer development, as in the example of the mutation of the JAK2 gene which induces alterations of the bone marrow environment (Arranz et al., 2014), or whether microenvironmental changes occur first (e.g. due to chronic inflammatory conditions) and are sufficient to initiate cancer development. It is also unclear how the tumour microenvironment in metastases relates to that in the primary tumour and whether changes in the tumour microenvironment are triggered by genetic or epigenetic events in the cancer cells (Marks et al., 2016; Taddei et al., 2013).

Therefore, the use of the eco‐evolutionary framework and the clear identification of the selective pressures in the tumour microenvironment will be of key importance to understand malignant cell development, dispersal and ability to evolve resistance to commonly used therapies.

#### 3.3.1 Questions

24. How does the microenvironment drive tumour progression?
25. What are the interactions between the tumour and its different environments?
26. How does ageing alter tissue microenvironments thereby selecting oncogenic cells?
27. How and when do cancer cells adopt different ‘foraging strategies’?
28. To which extent is tumour heterogeneity a cause or a consequence of oncogenesis?
29. What are the minimal essential resources necessary for cancer cell survival and can targeting them offer new therapeutic opportunities?
30. Given the diversity of the biological interactions inside neoplasms, to what extent does negative selection may operate during tumour evolution?
31. How to measure and quantify the reciprocal ecological and physiological feedbacks between host and tumours and their association with coping strategies?

### 3.4 Major theme 4: Infectious causes of cancer

It is well established that a significant proportion of human cancers, currently estimated to be around 20%, have an infectious causation (Dheilly et al., 2019; Ewald & Swain Ewald, 2014; zur Hausen, 2008). Infectious agents are known to abrogate barriers to cancer such as cell‐cycle arrest, apoptosis, telomerase regulation for non‐stem cells, cell adhesion for metastatic cancers and asymmetric division for stem cell cancers that block oncogenesis when they are in place (Ewald & Swain Ewald, 2014). In addition, infectious agents also disrupt processes that retard but do not block oncogenesis, such as restrictions of resources, vulnerability to immunological defences and regulation of cell division rates (Ewald & Swain Ewald, 2014, 2019). As a consequence, infectious agents can promote tumour formation and malignancy and cause the death of the host (Ewald & Swain Ewald, 2019; Plummer et al., 2016). It is likely that the proportion of cancers with infectious causations is currently being underestimated. For example, viruses can cause tumours in which only a low proportion of cells are infected (1% of tumour cells are Epstein–Barr virus‐positive) (Ewald & Swain Ewald, 2019). If a criterion based on a low viral load is used to rule out an infectious cause, it is therefore possible that the number of cancers with underlying infectious agents is being underestimated due to our limited understanding of how symbionts drive oncogenesis (Dheilly et al., 2019; Jacqueline et al., 2017). Another component that has not yet been integrated is the influence of nonparasitic symbionts on vulnerability to and protection from oncogenesis. If commensalism is considered to be a dividing line between mutualism and parasitism on the mutualism/parasitism continuum, this synthesis will involve an understanding of how mutualists and ambisymbionts (i.e. symbionts that can be parasitic or mutualistic depending on circumstance) may protect against oncogenic parasites, generate protective compounds and improve anti‐cancer immune functions. The use of an eco‐evolutionary framework is therefore required for understanding the joint contributions of parasites, mutations, environmental hazards and genetic vulnerabilities on cancer initiation and progression but also the influence of nonparasitic symbionts on vulnerability to and protection from oncogenesis (Dheilly et al., 2019).

#### 3.4.1 Questions

32. How many cancers have an infectious causation?
33. What are the interactive effects of symbionts (parasites, commensals and mutualists) as essential and exacerbating causes of cancer?
34. What are the ecological and environmental drivers affecting the emergence of infectious cancers?
35. What is the extent by which infectious agents can drive oncogenesis even if the cells infected by the agents represent only a small portion of the tumour cells?
36. What can be learned from the eco‐evolutionary approaches used to prevent and treat infectious diseases?

### 3.5 Major theme 5: Cancer and the immune system

Intracellular infections have contributed to the evolution of multiple immune checkpoints to cope with potential threats without self‐destruction. In 1893, Coley linked infection and cancer remission, and in 1970, Burnet proposed the important role of the immune system in policing cancer in his immunosurveillance hypothesis. Since these early works, the role of the immune system in cancer treatment has been demonstrated by the recent success of applying immunotherapy, particularly via using checkpoint inhibitors (Pardoll, 2012). For example, while resistance to proto‐oncogene B‐Raf (BRAF) inhibitors usually evolves in melanomas after only a few months of treatment, immunotherapy with checkpoint inhibitors, such as nivolumab and ipilimumab, is able to slow down the growth of these tumours, to induce durable immune responses and prolong survival (Larkin et al., 2019). In addition, mismatch repair‐deficient tumours (e.g. gastro‐oesophageal adenocarcinomas) are highly sensitive tumours to immunotherapy despite their extreme levels of genetic heterogeneity (von Loga et al., 2020). However, ambiguity remains on the role of immune system in cancer control. For example, the more recently developed immunoediting hypothesis, which postulates that through three stages (elimination, equilibrium and escape) the immune system iteratively selects for tumour cell variants with increasing capacities to survive and escape immune responses, is more pessimistic in assuming that tumour evolution breaks down initial immune system control (Dunn et al., 2002). Furthermore, only a subset of patients responds to immune checkpoint blockade in melanoma and lung cancers, and the critical features that determine response remain unclear (Koyama et al., 2016; Zaretsky et al., 2016). Previous studies have suggested that neoantigens deriving from somatic alterations (Rizvi et al., 2015), particularly those that are clonal in origin (Mcgranahan et al., 2016), may be principal targets for immune cells, such as CD8^+^ T cells. However, as every tumours present neoantigens that are unique to the cancer but also to the individual, developing broad‐spectrum and efficient immunotherapies remains a challenge. For example, compared with low heterogeneity tumours that present high clonal neoantigen burden, tumours with higher‐neoantigen heterogeneity may have a lower antigen dosage. This will further hinder treatment strategies as T cells reactive to specific subclonal neoantigens may be able to target only some, but not all cells in a tumour; moreover, identifying T cells reactive to very specific subclonal neoantigens can be a challenge (Rizvi et al., 2015). We also lack the full understanding of the temporal variation of immune responses to malignant cell development and progression throughout the life of an organism (as immune response to tumours is often measured only after cancer diagnoses, which tend to be made late during tumour development). More generally, we are still missing critical comparative analyses of immunosuppression across the Tree of Life. Overall, how the immune system actively sculpts tumour development and, reciprocally, how a patient's immune system is influenced by cancer evolution still remain unclear (Rosenthal et al., 2019). Also, from an evolutionary perspective, we cannot exclude the possibility that natural selection has adaptively optimized our immune system for only partially eradicate malignant cells (Thomas et al., 2018). Indeed, with the same logic than the one used in adaptive therapy (Gatenby et al., 2009), a restrained natural immune response would forestall immune‐resistant cancer cells and produce long‐term durable control of the cancer population.

#### 3.5.1 Questions

37. What are the roles of immunological checkpoints and tolerance in oncogenesis?
38. What are the key dynamics in the interactions of cancer cells and the host immune system?
39. What is the role of the immune system in shaping mutational landscapes and somatic evolutionary trajectories that lead to cancer?
40. How can we best harness a patient's immune system to tackle cancer evolution?
41. Why can immunotherapy (e.g. immune checkpoint inhibitors) seemingly cure even heterogeneous and rapidly evolving tumours against which other drug therapies rapidly fail due to resistance development and how we use these insights to design conventional therapies that are as effective?
42. Can we develop vaccines against early metastatic cells?
43. Does immune policing increase in large, long‐lived animals?

### 3.6 Major theme 6: Using eco‐evolutionary principles to improve existing cancer prevention and treatments

Cancers evolve in response to the selective pressures of our interventions. This implies that we will need to use principles from evolution (and ecology) to manage the ever‐evolving target of a cancer. A solid tumour is not simply a mass of cancer cells but is occupied by many interacting cell types (Barcellos‐Hoff et al., 2013). Cancer cells are ecosystem engineers, altering the ecosystem of the invaded healthy organ and able to evolve resistance to conventional therapies and develop into a hyperprogressive disease with consequences that lead to the death of the patient and of the cancer (Hansen et al., 2017; Sabio & Chan, 2019). Several factors such as population size, mutation mechanisms and rates as well as the strength of selection pressure are proposed to influence how rapidly a cancer cell population evolves (Lipinski et al., 2016; Salgia & Kulkarni, 2018). In the last 10 years, single‐cell analyses have also provided key evidence for the importance of non‐genetic heterogeneity in cancer evolution and drug resistance (Navin et al., 2011). Especially, phenotypic plasticity produced by gene expression variability has been associated with important phenomena such as persister cells (Ramirez et al., 2016), apoptosis (Spencer et al., 2009), stemness (Patel et al., 2014) or metastasis (Nguyen et al., 2016). At the same time, next‐generation sequencing and cancer genome programmes revealed the degree of genetic inter‐ and intratumoral heterogeneity and fostered our understanding of tumour evolution at the genetic level (Burrell et al., 2013), see above. In order to design effective cancer therapies, we need to know the proportion of subpopulations of cancer cells differing in their resistance mechanisms. For example, there are three cell types in metastatic castrate‐resistant prostate cancer populations: cells dependent on testosterone, cells that are able to produce testosterone and cells independent of testosterone (Zhang et al., 2017). For other cancer types, no such clear cancer cell subtypes have yet been identified, and it may be that each cell has a potential for resistance as a continuous and evolving trait instead. While we may be able to estimate the approximate tumour composition from relevant biomarkers combined with volumetric information (Alix‐Panabières & Pantel, 2014; Pantel & Alix‐Panabières, 2019; Staňková, 2019), this may not be precise enough in some cancers to guide therapies. For metastatic diseases, biopsies cannot be sampled frequently enough and may not give us relevant information for all tumour sites. The way forward may be liquid biopsies (e.g. using circulating tumour cells and cell‐free DNA; Crowley et al., 2013). However, even then we will probably get averaged information on cell types within the patient's body and not on the state of cancer cells within each of the tumour sites. This will be of concern if there is a high diversity among these different sites. In the coming years, the main challenge will be to integrate these various types of heterogeneity in a global picture of cancer evolution and to consider the respective influence of genetic or/and non‐genetic heterogeneity in the different steps of the oncogenesis process. This is a key step in designing efficient therapies and reducing the likelihood that a tumour will evolve resistance to drug treatments. Its importance is evidenced by the tendency for different patients to show dramatically different responses to the same treatment (Sun & Yu, 2015). This integrated picture should allow researchers to identify when and why treatment resistance can be reversed, allowing certain drugs to be reused. The PCAWG Consortium revealed an unprecedented scale of cancer complexity and thus highlighted the gargantuan obstacles ahead in cancer treatment (Campbell et al., 2020).

Another challenge will be to reassess the language and metaphors we use for cancer, and these denominations also influence the way we treat cancer and how we care for patient. The war metaphor, for example, positions us in opposition to cancer in a way that can lead to ineffective prevention measures (Hauser & Schwarz, 2015), and possibly bias us towards overly aggressive treatments (Aktipis et al., 2011). Because cancer is an evolving population that can respond to our treatments, and an evolutionary foe we have lived with since the origins of multicellularity, we need to find appropriate metaphors that take into account those facts and help us think about effective ways of approaching cancer (Aktipis, 2020).

#### 3.6.1 Questions

44. Which ecological and evolutionary principles can be applied to slow down somatic evolution and prevent or slow down cancer progression?
45. When is it best to aim for tumour elimination and when for containment?
46. Can we influence the ability of cancers to evolve in order to delay, reduce or stop acquisition of drug resistance?
47. How do genetic and non‐genetic heterogeneities impact cancer evolution and drug resistance?
48. Can evolution of resistance be reversed?
49. How can different mechanisms of resistance influence treatment prospects?
50. What is the best treatment choice based on the speed of evolution of resistance in cancer cells?
51. How can we estimate accurately the eco‐evolutionary state, resistance level and tumours heterogeneity in vivo?
52. How can we select a treatment that addresses all heterogeneous tumour sites within one patient?
53. What is the contribution of cellular plasticity (as opposed to mutational change) to cancer adaptation and how central is phenotypic plasticity in cancer and drug resistance during tumour progression and drug treatment?
54. How can we exploit cooperative ecosystem engineering to expose unique and targetable vulnerabilities of the tumour ecosystem?
55. To what extent comparative oncology can help to identify novel solutions for cancer treatments?
56. Can we effectively prevent cancer mortality by intervening with the proximal causes of cancer death (e.g. cachexia, cytokine storms)?
57. How exactly do different cancer cells compete with each other, and can this mechanism of competition be enhanced by therapy (e.g. adaptive therapy)?
58. What proportion of cancer is preventable by lifestyle modifications and how can we aid in the social change to implement these interventions?
59. How is the trait of evolvability selected for in the tumour ecosystem and how does it change our understanding of cancer cell evolution?

### 3.7 Major theme 7: Conceptual and mathematical models of cancer development and outcomes

Mathematical modelling of cancer has been expanding in the field of cancer ecology and evolution as a potentially valuable tool to complement experimental research (Archetti & Pienta, 2019; Dhawan et al., 2016; Maley et al., 2017). Indeed, with the vast quantities of information that are currently generated, and with a vast number of conditions and hypotheses to be tested, including computational tools for such work can be indispensable. Mathematical modelling involves formalizing assumptions about biological processes and describing them in terms of either equations (classical mathematical modelling that calculates solutions to these equations subject to specific parameter values and initial conditions) or rules (agent‐based modelling/simulations, analysed as in silico experiments with elements of stochasticity) (Altrock et al., 2015; Anderson & Quaranta, 2008; Beerenwinkel et al., 2015; Bellomo et al., 2008). Mathematical models are powerful tools that can help both organize understanding of the biology, test hypotheses and identify gaps in knowledge, since a model will predict what will happen if the underlying assumptions hold (and if they do not match observations, then a gap in knowledge has been identified). Promising avenues include schemes based on evolutionary and ecological indices, such as applying game theory and Lotka‐Volterra equations to cancer treatment and other mechanistic models that recapitulate evolutionary dynamics or network models that investigate gene interactions (Archetti & Pienta, 2019; Dhawan et al., 2016; Mair et al., 2019; Maley et al., 2017). However, it is not yet established whether such models can outperform standard prognostic methods, nor do we know exactly what data types are needed for forecasting. Patients can show dramatically different responses to the same treatment and identifying the correct biomarkers will assist in developing models to understand how individual patients will best respond to different therapies. Those models will then be used to maximize survival chances and minimize the risk of the cancer evolving into a hyperprogressive disease (where the treatment accelerates the progression of the cancer) (Hansen et al., 2017; Sabio & Chan, 2019). Furthermore, we need to remember that models are also as good as the assumptions that went into them, and thus, useful models should be created in collaboration with biologists and experimentalists.

#### 3.7.1 Questions

60. Can we forecast a tumour's next evolutionary step?
61. Is the genetic model of carcinogenesis correct and do we need to develop alternative models to improve our ability to forecast tumour evolution?
62. How can game theory be utilized to understand tumorigenesis and potentially guide therapy?
63. Are there measures of the evolution and ecology of tumours that can be used to develop a classification system for tumours, so as to improve prediction, prognosis and management of tumours?
64. To what extent do the widely used model systems in cancer research represent the ecological and evolutionary processes governing tumour emergence and progression and how can comparative oncology be used to find new research directions?
65. How can tumour ecology be used to improve the search for biomarkers and predict patient outcomes?
66. What lessons can we learn from the evolutionary dynamics of species extinction for cancer therapy?

### 3.8 Major theme 8: Species‐specific strategies for cancer prevention

Cancer is a disease that arose with the evolution of multicellularity and has been a major force shaping ecological and evolutionary processes in wildlife populations (Aktipis & Nesse, 2013; Thomas et al., 2017). Multicellular organisms evolved to resolve conflicts between individual cells and protect the internal organization of the individual by using cancer suppressor systems. In many aspects, tumours can be viewed as new biological entities, with rapidly expanding genetic diversity, that are no longer integrated in the functioning of the host organism, especially in the original local microenvironment (Egeblad et al., 2011). This especially applies to transmissible cancers. While currently rare, there is still a major open question about how common transmissible cancers may have been in the evolution of species (Ujvari et al., 2016b). It is possible that they were much more common earlier during the evolutionary history of life on earth, and that species simply evolved mechanisms for preventing and suppressing potentially transmissible cancers, explaining the low number of extant transmissible cancers (Aktipis, 2020; Ujvari et al., 2016a). It is also likely that the evolution of humans has been shaped by cancer. Despite an increasing cancer incidence due primarily to lifestyle changes and ageing populations, the majority of people live without life‐threatening cancer their whole life (Bissel & Hines, 2011). While precursor lesions or carcinoma in situ are found in a considerable amount of individuals, it is still poorly understood how resistance mechanisms evolved to constrain expansion of oncogenic cells or tolerance by which the lesion or carcinoma is able to reduce their fitness (Thomas, Giraudeau, Gouzerh, et al., 2019; Thomas, Giraudeau, Renaud, et al., 2019). Genetic endowment seems to determine how the organism copes with harmful extrinsic (e.g. tobacco smoke, UV) and intrinsic (e.g. obesity) factors, how these modulate the host's tissues (inducing local low‐grade inflammation or not) and whether the combination of these factors leads to oncogenic events and cancer evolution (Dujon, Ujvari, et al., 2020; Pham‐Danis & DeGregori, 2019; Rozhok & DeGregori, 2015). Thus, survival, selection and expansion of transformed cells apparently depend on the microenvironmental context given by the quality and quantity of damaging factors, duration of exposure and host genetics. Hence, a better understanding of the host genetics conferring resistance and/or tolerance to cancer is urgently needed. Prevention and treatment strategies should aim at maintaining tissue homeostasis to impair selection for oncogenic clones.

#### 3.8.1 Questions

67. What is the importance of cancer in ecosystem functioning?
68. To what extent should oncogenesis be considered as a speciation process?
69. What is the relevance of tumorigenesis as a selective force in nature and how does it shape ecological and evolutionary dynamics across species?
70. How have other species evolved to reduce the risk of developing cancer (e.g. naked mole rat, elephants), and can we translate those to human cancer prevention?
71. Which host‐related factors are key determinants for conferring tolerance to cancer evolution?
72. What was and is the role of humans in causing cancers in the wild? Do host populations evolve resistance to transmissible cancers, or can cancers evolve to become less pathogenic over time?
73. How do rapid environmental changes such as global warming, increased exposure to novel pathogens and toxins contribute to species cancer risk?
74. Will the daily exposure to pesticides by humans and wildlife increase drastically the prevalence of cancer within the next decades?
75. How will the evolution of the human species (driven by contemporary aspects such as changes in environment and lifespan) affect the impact of cancer on human populations, and conversely, how will cancer impact the evolution of human species?
76. What can we learn from people who have lived long cancer‐free lives, including those exposed to mutagens, to understand what makes them resistant to cancer?
77. How have transmissible and non‐transmissible cancers contributed to the evolution of species on the planet?
78. Are transmissible cancers under continued selection for novelty (positive selection) due to genetic conflict with their host or are they under selection for conservation (negative selection), or are they simply selectively neutral?
79. Is somatic evolution driven by mutations or natural selection?

### 3.9 Major theme 9: Obtain insights from wild species

Considering that cancer is present in most metazoan species, comparing how various species have responded to the fitness reducing effect of cancer over the aeons of evolution (with the help of comparative oncology and evolutionary ecology) opens the opportunity for this knowledge to be translated to human cancer therapy (Albuquerque et al., 2018). These approaches include deciphering why species under significant environmental stress do not develop cancer (including long‐lived humans, as mentioned above). This is a challenging task because cancer is difficult to detect in wildlife species and requires the development of new biological markers, and cancer risk factors can be difficult to quantify (Dujon, Ujvari, et al., 2020; Hamede et al., 2020). Apart from multicellular organisms, insights can also be obtained from bacteria and other unicellular organisms even if they do not develop cancer. For example, the dynamic field studying the influence of phenotypic heterogeneity on treatment outcomes in cancer has largely been inspired by works on microorganisms that have demonstrated how gene expression variability and the associated cell‐to‐cell heterogeneity can produce subpopulations with distinct behaviours of non‐genetic origin (Ackermann, 2015). In addition, striking similarities have been observed between the appearance of subpopulations tolerant to environmental stress in microbial populations (Blake et al., 2006) and cell responses to therapeutic pressure in cancer cell populations (Shaffer et al., 2017).

Finally, although currently considered to be rare (Ujvari et al., 2016b) but see Dujon et al. (2020), transmissible cancers present as inter‐individual metastases (Dujon, Gatenby, et al., 2020) and hence can provide valuable insights in order to curtail human cancer cell progression and dispersal. Although the conditions that allow transmissible cancer lineage emergence and persistence are not fully understood, these intriguing clonal infectious cell lines (that act as cancer‐causing infectious agents) use similar mechanisms and pathways to avoid immune recognition and elimination as human cancers. Translating the information about their capacity to overcome challenges in and across hosts (immune recognition, survival in transit, etc.) could contribute to novel treatment strategies of metastatic cancers and malignancies with underlying infectious aetiologies. In addition to human cancer research and treatment, comparative oncology can also significantly contribute to the conservation of species in which cancer is a concern (e.g. Tasmanian devils or sea turtles) (Hamede et al., 2020). Furthermore, transmissible cancers offer excellent examples of how scientists organized themselves into efficient collaborative and multidisciplinary networks to obtain insights on those diseases (Dujon, Bramwell, Raven, et al., 2020).

#### 3.9.1 Questions

80. How much will the biology of microorganisms inform and guide cancer research?
81. Which are the conditions allowing transmissible cancer lineages to start and spread (and can it happen in humans)?
82. What can we learn from long‐lived animals, or animals that are exposed to excess oxidative damage, UV radiation, but rarely develop cancer?
83. What are the predictors (life history, physiology, environment, etc.) of interspecific differences in cancer prevalence and how can comparative oncology help to initiate new lines of research for cancer treatments?
84. How do we identify and develop informative cancer biomarkers for non‐human species?

## 4 CONCLUDING REMARKS

By assembling the major challenging questions and placing them into specific scientific context, our objective was first to broaden the portfolio of research directions and methods to stimulate novel approaches and progress at the interface of oncology and ecological and evolutionary sciences. In addition to highlighting what we know, what we do not know, and where we should focus our research and practice, several general conclusions can be drawn from this summary. First, it is clear that the previous traditional separation of scientific disciplines with different perspectives on the same biological problem urgently needs to be overcome in order to make headways in our understanding of complex processes, such as the evolutionary ecology of host–tumour interactions. We believe that the responses to many current and future questions about cancer will come as a result of progressive and productive multidisciplinary collaborations that draw on the insights and the expertise of multiple scientific fields. A few (so far rare) examples of successful collaborations already exist, for example collaboration between mathematicians, ecologists and clinical oncologists spearhead revolutionary cancer treatment strategies that successfully incorporate evolutionary dynamics into cancer therapy at the Cancer Biology and Evolution Program, Moffitt Cancer Center, Tampa, Florida (Gatenby & Brown, 2020). Similarly, the Cancer and Evolution Laboratory at the Arizona State University is researching fundamental concepts in neoplastic progression and therapeutic resistance (Martinez et al., 2018) and the Cancer Ecology and Evolution international laboratory (between Deakin University, the University of Tasmania in Australia and the Centre de Recherches Écologiques et Évolutives sur le Cancer in France) focuses on understanding the ecological and evolutionary consequences of cancer in ecosystems (Dujon, Ujvari, et al., 2020; Giraudeau et al., 2018). Second, despite major progresses, especially in recent years, the topic of Ecology, Evolution and Cancer is still in its infancy, and a much larger global research effort is required. We believe that the questions compiled, and the directions outlined in this paper will stimulate further discussions, open up avenues for novel prevention and treatment approaches. Although ecological and evolutionary principles have already provided novel insights into several cancer‐related topics, by identifying the major themes across the crossroads of evolutionary and cancer biology, we provide a focused guideline for future research.

## GLOSSARY

**Neoplasia**: New, uncontrolled growth of cells that is not under physiologic control.

**Life‐history trait**: Term used in evolutionary ecology sciences to describe a species' or population's reproductive strategies. It concerns parameters such as the number, size and sex ratio of offspring, the reproduction timing, age and size at maturity and growth pattern, longevity and ageing. Combinations of these life‐history traits create the life‐history strategies. Life‐history strategies evolve by natural selection, being an optimization of trade‐offs between growth, survival and reproduction.

**Commensalism**: A biological interaction in which individuals of one species gain benefits, while those of the second species neither benefit nor are harmed.

**Eco‐evolutionary**: The unidirectional effects of ecological changes in evolutionary processes or the unidirectional effects of evolutionary changes in ecological processes.

**Parasitism**: A biological interaction in which individuals of one species gain benefits, while those of the second species are harmed.

**Mutualism**: A biological interaction in which individuals from each species have a net benefit.

**Inclusive fitness**: Taking into account not only the reproductive success of an individual or a cancer cell, but also its effects on the survival and reproductive success of its kin.

**Immune checkpoint inhibitors**: A type of immunotherapy drug that blocks immune checkpoints, which are biochemical mechanism that help to keep immune responses from being too strong. Examples of checkpoint proteins found on T cells or cancer cells include PD‐1/PD‐L1 and CTLA‐4/B7‐1/B7‐2. When these checkpoints are blocked, T cells can kill more cancer cells.

## CONFLICT OF INTEREST

The authors declare no conflict of interest.

## Supporting information

Appendix S1

## Data Availability

The data that support the findings of this study are available in the supplementary material of this article.
