# Supplementary material for: Identifying key questions in the ecology and evolution of cancer
Source: Evol Appl. 2021 Feb 8;14(4):877–92. doi: 10.1111/eva.13190 (PMC8061275; doi:10.1111/eva.13190)
Supplement: Supplementary file 1 — Appendix S1 [file EVA-14-877-s001.docx]

**Identifying key questions in the ecology and evolution of cancer – Supplementary Material**

**Appendix S1. List of questions provided by the authors of: “Key questions in the ecology and evolution of cancer**”

- Which genetic aberrations acquired in subclones during cancer progression confer a fitness advantage, what phenotypes do these establish and which selection pressures promote their occurrence?
- Transmissible cancer: Do host populations evolve resistance to transmissible cancers, or can cancers evolve to become less pathogenic over time?
- What is the core eco-evolutionary program that manifests independently in cancer patients that leads to death?
- Why do malignant cells metastasize?
- Which host-related factors are key determinants for conferring resistance and tolerance to cancer evolution?
- How can we best forecast tumour evolution?
- How have recent studies describing mutational and somatic evolutionary processes in normal tissues changed our understanding of aging and the evolution of tumor suppression?
- What is the respective role of genetic and non-genetic heterogeneity in cancer evolution and drug resistance?
- How can early cancer driving mutations be rSNP loci and predicted neoantigens – why are these not eliminated from the gene pool in general and why are they not eliminated when tumors start to evolve (in people who do not have them as rSNP but are early mutations during tumor initiation)?
- How can we control (or prevent) therapeutic resistance?
- How does rapid environmental change (i.e. evolutionary mismatch), including loss/fragmentation of local habitats, global warming, increased exposure to novel pathogens and toxins, contribute to organismal cancer risk?
- What (additional) evolutionary principles can be applied to prevent or slow-down the emergence of resistance and/or overcome its effects during cancer therapies?
- How to estimate accurately the eco-evolutionary state (incl. tumor composition/resistance levels) of tumors in vivo?
- What are the interactive effects of symbionts (i.e., parasites, commensals, and mutualists) as factors in essential and exacerbating causes of cancer?
- When does a clone become a malignancy?
- How can the ecology and evolution of tumors be exploited to improve cancer treatment?
- Is somatic evolution driven by mutations or Natural Selection?
- Is the genetic model of carcinogenesis correct?
- What are the ecological characteristics of the tumor that induce dispersal from the primary tumor?
- What are the predictors (life history, physiology, environment…) of the differences observed in cancer prevalence between species and the applications of the comparative oncology to initiate new lines of research to develop cancer treatments?
- How do tumours metastasize?
- Why not all cancers are malignant? What’s the difference between benign and malignant tumors? Can we select for malignant cells to evolve into benign cells?
- What is the relevance of tumorigenesis as a selective force in nature and how it shapes ecological and evolutionary dynamics across species?
- What are the mechanisms by which natural selection increases cancer suppression in large long-lived animals?
- What strategies can we use to best keep cancer under control in the clinic so that patients can live with it rather than dying of it?
- Is cancer the origin of a new single cell protist?
- What is the contribution of cellular plasticity (as opposed to mutational change) to cancer adaptation during tumour progression and drug treatment?
- Transmissible cancer: What are the conditions which allow transmissible cancer lineages to start and spread (and can it happen in humans)?
- Why do cancer cells metastasize?
- Are there particular life periods where we are especially vulnerable to cancer initiation and why?
- Why do oncogenic cells disperse early from the primary site (and small lesions) ?
- Which mechanisms explain variation in cancer risk, relative to lifetime number of stem cell divisions?
- What is the role of the immune system in shaping mutational landscapes and somatic evolutionary trajectories (leading to cancers or not)?
- How central is phenotypic plasticity in cancer?
- How can mutant clones expand in normal tissues? Do they have higher fitness or it’s just a random process or part of cell competition in normal tissues? Why is there no immune response against them? Or are they mostly non-antigenic or rSNPs, so there is tolerance for them? But, then why are some of these mutants presented as neo-antigens as cancer evolves? What roles the clonal expansion of mutant clones in histologically normal tissues play in immune escape?
- Are there measures of the evolution and ecology of neoplasms that can be used to develop a classification system for neoplasms, so as to improve prediction, prognosis and management of neoplasms?
- How have other species solved the problem of cancer?
- Can we use cancer’s intrinsic evolutionary vulnerabilities to design more efficient anti-cancer drugs and therapeutic strategies?
- How to evaluate tumor heterogeneity and select a treatment that addresses all the heterogeneous sites within one patient?
- What are the roles of immunological checkpoints and tolerance in oncogenesis?
- Can we perform an extensive multilayer evaluation of intrinsic factors involved in clonal cancer evolution?
- How can tumor ecology be used to improve the search for biomarkers and predicting patient outcomes?
- How to reconcile considerations of cancer progression from evolutionary and developmental paradigms?
- Can evolution of resistance be delayed or stopped.
- How and when do cancer cells adopt different foraging strategies?
- The importance of cancer in ecosystem functioning and how human activities might impact it?
- Can we forecast a tumour’s next evolutionary step?
- Two out of three people never develop cancer, including committed smokers; strongly suggesting that evolution has resulted in effective cancer resistance mechanisms. Why are we not focusing on people who lived long, cancer free lives, to try to understand what makes certain people resistant to cancer?
- How does host phenotypic plasticity in response to oncogenic processes affects the evolutionary trajectory of cancer cells?
- What are driver mutations and how to identify them from cancer genomes?
- Our bodies are made up of 30 trillion highly cooperative cells that make us viable as multicellular organisms, and cancer occurs when this cooperation breaks down. This means that it is important to know: How do our bodies detect cellular cheating (i.e., potentially cancerous cells) and respond to that cheating effectively so that we do not succumb to cancer?
- Does cancer occur in three distinct phases of initiation, trade-off free, and finally tradeoff limited evolution?
- What is the role of microenvironmental change in promoting cancer progression and drug resistance and is this controlled by the cancer cells?
- Transmissible cancer: Are transmissible cancers under continued selection for novelty (positive selection) due to genetic conflict with their hosts, or are they under selection for conservation (negative selection), or are they simply dividing with neutral selection.
- How can eco-evolutionary dynamics help us understand how cancer cells respond and evolve in response to the stresses of the tumor microenvironment?
- How many cancers have an infectious causation?
- How does aging alter tissue microenvironments thereby selecting oncogenic cells?
- When is it best to aim for tumour elimination and when for containment?
- To what extent do cancers limit the fitness of wild animal populations, and where a clear impact is evident, what role did humans play?
- Should genetic intratumoral heterogeneity encourage new research avenues?
- Is tumor progression largely a host/microenvironment (local and systemic, including immune) driven process? If so, can we identify patients at diagnosis who are more/less likely to progress and target this progression-driving events at diagnosis?
- Can we prevent cancer (and improve therapeutic control of cancer) by slowing cancer evolution?
- hat are the necessary and sufficient conditions for cancer cells to successfully metastasize?
- How can we use ecological principles to suppress the initiation of the metastatic process, or decrease the metastatic potential of cancer cells in transit and their likelihood to establish new tumours?
- How different mechanisms of resistance influence treatment prospects?
- What is the extent to which infectious agents can drive oncogenesis even if infectious cancerous cells comprise only a small portion of the tumor cells?
- What are the interactions between the tumor and its environments?
- How should drug-discovery and clinical trials be modified to support development of eco-evolutionary approaches to treatment?
- How to integrate different sources of phenotypic variability towards inclusive inheritance in somatic evolution?
- Is extinction a viable conceptual model of cancer therapy
- How is the trait of evolvability selected for in the tumor ecosystem and how does it change our understanding of cancer cell evolution (and treatment strategies)?
- The development of new biomarkers of cancer for non-human species?
- To what extent does negative selection operate during tumour evolution?
- Most cancer related deaths are caused by metastatic cancers. Can we develop vaccines against (early) metastatic cells, by using their epigenetic/cellular/phenotypic profiles?
- How to measure and quantify the reciprocal ecological and physiological feedbacks between host and tumours and their association with coping strategies such as cancer tolerance and resistance?
- Does immune policing increase in large, long-lived animals?
- How do the language and metaphors we use for cancer influence the way we treat cancer and how we care for patients?
- Does cancer evolution progress towards additional speciation involving niche filling and partitioning?
- Can we influence the ability of cancers to evolve in order to delay drug resistance acquisition?
- Transmissible cancer: Is mitochondrial replacement in CTVT and possibly BTN a common feature of transmissible cancers (and perhaps human cancers as well), and is there a benefit to the cancer, the mitochondria, or neither?
- Can eco-evolutionary principles drive and direct cancer therapy?
- To what extent comparative oncology can help to identify novel solutions for cancer treatments?
- How can the highly variable intratumor heterogeneity be explained and how does it contribute to evolution of distinct tumors?
- What are the true cancer drivers?
- What mechanisms account for tumor heterogeneity, and how could the generation of such heterogeneity be controlled?
- How much the biology of microorganisms will inform and guide cancer research?
- Related to this: what is the difference between people who develop aggressive tumors at young age vs. indolent tumors (including those that never get diagnosed in the clinic) at older age? What degree is this due to age-related differences in the host/microenvironment? Could we learn something related to this from pediatric tumors that’s relevant for adult cancers as well or pediatric tumors are almost always due to some germline effects (polymorphisms or mutations)?
- What mechanisms has organismal evolution discovered to prevent cancer, and can we translate those to human cancer prevention?
- Can we treat cancer a chronic disease by using evolutionary and ecological principles and what are the best evolutionary and ecological predictors for patient to guide treatment strategies?
- What ecological and evolutionary principles can be applied to slow down somatic evolution and prevent or slow down cancer progression?
- What is the role of the tumor microenvironment during forming metastases?
- What are the ecological and evolutionary interactions between cells and both micro- and macro-environments that contribute to oncogenesis?
- What is the cell of origin in cancer?
- What can be learned from the eco-evolutionary approaches used to treat infectious disease?
- Can we improve therapeutic outcomes through explicit consideration of principles from ecology and evolution?
- Can normal somatic cells evolve?
- What are the minimal essential resources necessary for cancer cell survival?
- Why do some organs develop on average cancer before others?
- How do epistatic interactions shape cancer development?
- What can we learn from long-lived animals, or animals that are exposed to excess oxidative damage, UV radiation but rarely develop cancer?
- To what extent the widely used model systems in cancer research represent the ecological and evolutionary processes governing tumour emergence and progression and how comparative oncology can be used to find new research directions?
- How can we use evolutionary theory to determine how much cancer risk results from the modern human environment?
- How many of our cancer suppression systems may have (at least partially) evolved to keep transmissible cancer at bay?
- Are most cancer cells most of the time at or near eco-evolutionary optima?
- Why can the immunotherapy seemingly cure even heterogeneous and rapidly evolving tumours against which other drug therapies are ineffective because of resistance development and can we learn from this how to make cancer therapies more effective overall?
- Adaptive Therapy: How exactly do different cancer cells compete with each other, and can this mechanism of competition be enhanced by therapy?
- How can game theory be utilized to understand tumorigenesis and potentially guide therapy?
- What is the contribution of malignant cells to inter-individual variability and ecosystem functioning?
- How does division of labour drives cancer evolution?
- What is a useful model of the eco-evolutionary dynamics of early metastatic development?
- How can an evolutionary understanding of cancer be exploited for detecting, preventing and treating cancers?
- To what extent oncogenesis should be considered as a speciation process?
- Are tumors with the same epigenetic state but different tissue of origin really initiates from different tissue-specific progenitors or could they actually originate from the same cell type that’s located in different organs or even circulating? For example, neuroendocrine tumors of different origin (prostate, lung) look epigenetically very similar. How can we prove cell-of-origin in humans??
- Can we effectively prevent cancer mortality by intervening on the proximal causes of cancer death (e.g., cachexia, cytokine storms, etc.)?
- What proportion of cancer is preventable by lifestyle modifications and how can we aid in the social change to implement these interventions?
- How will the evolution of the human species (driven by contemporary aspects such as changes in environment and lifespan) will affect the impact of cancer on human populations, and, conversely, how will cancer impact the evolution of human species?
- What is the best treatment choice based on the speed of evolution of resistance in cancer cells?
- What is the extent to which tumor heterogeneity a cause or a consequence of oncogenesis?
- What is the role of intra-clonal competition or cooperation?
- Can eco-evolutionary theory be used to predict when treatment resistance is reversible?
- Is inclusion of ecological perspective required to understand initiation and progression of cancers?
- What are the key dynamics in the interactions of cancer cells and the host immune system?
- How can we exploit cooperative ecosystem engineering to expose unique and targetable vulnerabilities of the tumor ecosystem?
- Will the daily consumption of pesticides by most humans (and wild animals) increase drastically the prevalence of cancer within the next decades?
- How can we best harness a patient’s immune system to tackle cancer evolution?
- How did cancer/malignant cells (transmissible and non-transmissible as well as those that are caused by infectious agents) have contributed to the evolution of organisms on the planet?
- What are the ecological and environmental drivers affecting the emergence of infectious cancers (viral and non-viral)?
- What are the conditions necessary for the establishment of a transmissible cancer?
- How can we most effectively utilize tools from evolutionary biology and ecology to diagnose and treat cancer?
- Using evolutionarily enlightened therapies do we have all of the therapeutics needed to cure most cancers at most stages?
